# Trichloroethylene Cancer Epidemiology: A Consideration of Select Issues

**DOI:** 10.1289/ehp.8949

**Published:** 2006-05-09

**Authors:** Cheryl Siegel Scott, Weihsueh A. Chiu

**Affiliations:** National Center for Environmental Assessment, Office of Research and Development, U.S. Environmental Protection Agency, Washington, DC, USA

**Keywords:** cancer, drinking water exposures, epidemiology, occupational exposures, risk assessment, trichloroethylene

## Abstract

A large body of epidemiologic evidence exists for exploring causal associations between cancer and trichloroethylene (TCE) exposure. The U.S. Environmental Protection Agency 2001 draft TCE health risk assessment concluded that epidemiologic studies, on the whole, support associations between TCE exposure and excess risk of kidney cancer, liver cancer, and lymphomas, and, to a lesser extent, cervical cancer and prostate cancer. As part of a mini-monograph on key issues in the health risk assessment of TCE, this article reviews recently published scientific literature examining cancer and TCE exposure and identifies four issues that are key to interpreting the larger body of epidemiologic evidence: *a*) relative sensitivity of cancer incidence and mortality data; *b*) different classifications of lymphomas, including non-Hodgkin lymphoma; *c*) differences in data and methods for assigning TCE exposure status; and *d*) different methods employed for causal inferences, including statistical or meta-analysis approaches. The recent epidemiologic studies substantially expand the epidemiologic database, with seven new studies available on kidney cancer and somewhat fewer studies available that examine possible associations at other sites. Overall, recently published studies appear to provide further support for the kidney, liver, and lymphatic systems as targets of TCE toxicity, suggesting, as do previous studies, modestly elevated (typically 1.5–2.0) site-specific relative risks, given exposure conditions in these studies. However, a number of challenging issues need to be considered before drawing causal conclusions about TCE exposure and cancer from these data.

Despite numerous reviews [Brüning and Bolt 2000; International Agency for Research on Cancer (IARC) 1995; Institute of Medicine 2002; Lynge et al. 1997; McLaughlin and Blot 1997; National Toxicology Program (NTP) 2002; Wartenberg et al. 2000; Weiss 1996; Wong 2004], including those of two multidisciplinary expert panels that concluded that trichloroethylene (TCE) is “probably” (IARC 1995) or “reasonably anticipated to be” (NTP 2002) carcinogenic in humans, the interpretation of the epidemiologic studies on cancer and TCE exposure remains an area of considerable debate. The strongest epidemiologic evidence for associations between TCE exposure and cancer is for liver cancer, kidney cancer, and lymphomas, but perspectives have differed about the causal inferences regarding the human carcinogenicity of TCE that can be drawn from the epidemiologic database as a whole (e.g., Mandel and Kelsh 2001; Wartenberg et al. 2000). Some of the key issues underlying different interpretations are the use of different qualitative and quantitative (e.g., meta-analysis) methods to synthesize the body of evidence and the weight given to studies on the basis of different measures of cancer risk (e.g., incidence versus mortality) and different methods of exposure assessment. In addition, interpretation of data on lymphomas poses unique challenges because of the use of different classification systems and an evolving understanding of their etiology. As discussed in the overview article on this mini-monograph (Chiu et al. 2006a), these are all issues on which the National Academy of Sciences (NAS) has been asked to provide advice.

In this review we first summarize the recent epidemiologic literature on TCE exposure and cancer occurrence and then discuss the issues identified above as key to interpreting the larger body of epidemiologic evidence. Although some scientific conclusions can be drawn from this updated body of data, speculation about the impact of these data on the final TCE risk assessment would be premature at this point, given the ongoing NAS consultation discussed in the overview article by Chiu et al. (2006a) and the planned revision of the U.S. Environmental Protection Agency (EPA) TCE risk assessment. Therefore, the purpose here and throughout this mini-monograph is to review recently published scientific literature in the context of how it informs the key scientific issues believed to be most critical in developing a revised risk assessment.

## Epidemiologic Studies on Cancer and TCE Exposure

The epidemiologic analysis in the U.S. EPA draft TCE risk assessment (U.S. EPA 2001) was supported in large part by the review by Wartenberg et al. (2000). This review identified more than 80 studies that evaluated cancer and TCE exposure, concluding that the evidence more firmly supported associations of TCE exposure with kidney and liver cancer while providing some support for associations with non-Hodgkin lymphoma (NHL). Wartenberg et al. (2000) also noted possible associations between TCE exposure and multiple myeloma and prostate, laryngeal, and colon cancer as well as cervical cancer and TCE or perchloroethylene exposure.

A number of studies and literature reviews have been published since 2000. Tables 1–3 provide short descriptions of these studies, which include historical or retrospective cohort studies (Table 1), case–control studies (Table 2), and ecologic or community studies (Table 3). Most of the TCE cohort and case–control studies involve occupational exposure to TCE, primarily by inhalation, whereas community studies usually involve contaminated groundwater where potential TCE exposure may be through both ingestion of drinking water and inhalation from TCE vapor intrusion into sub-surface residential areas or from showering. Many of these studies employed more sophisticated exposure assessment approaches, allowing better identification of likely TCE-exposed subjects (Brüning et al. 2003; Charbotel et al. 2006; DeRoos et al. 2001; Dumas et al. 2000; Hansen et al. 2001; Pesch et al. 2000a, 2000b; Raaschou-Nielsen et al. 2003; Zhao et al. 2005). Tables 4–7 show corresponding study results for cancers that either are newly reported to have associations (Table 4, total cancers and cancers of the bladder, breast, and esophagus) or have drawn the most attention in previous reviews [Table 5, kidney cancer or renal cell carcinoma (RCC); Table 6, cancer of the liver or liver and biliary passages; Table 7, lymphomas]. These recent studies substantially expand the epidemiologic database, providing additional insights on potential causal associations between TCE exposure and cancer occurrence. The following discussion focuses on the three groups of end points—kidney cancer and RCC, liver and biliary cancer, and lymphomas—previously identified as having the strongest evidence for potential causal association with TCE exposure (IARC 1995; NTP 2002; Wartenberg et al. 2000).

The studies available since 2000 report consistent associations between kidney cancer or RCC and TCE exposure (Table 5). Two cohort studies with large numbers of exposed cases (Raaschou-Nielsen et al. 2003; Zhao et al. 2005) observed statistically significant associations with greater exposure level or duration of employment. These findings were supported by three recent case–control studies assessing TCE exposure in the metal industry in Germany (Brüning et al. 2003; Pesch et al. 2000a) and in France (Charbotel et al. 2006). The studies by Brüning et al. (2003) and Charbotel et al. (2006) were designed specifically to examine the *a priori* hypothesis of an association between RCC and TCE exposure. Charbotel et al. (2006) suggested that exposure intensity may contribute to the risk associated with cumulative exposure because risks were higher for subjects in the highest cumulative exposure category with peak TCE exposure [odds ratio (OR) = 2.7; 95% confidence interval (CI), 1.1–7.1] than for subjects with only high cumulative exposure (OR = 2.2; 95% CI, 1.0–4.6), compared with unexposed subjects.

Most of the recent cohort studies also provide information as to possible association between TCE and liver and/or biliary tract cancer, although many examined only the combined category (Table 6). Grouping the adjacent, but anatomically distinct, end points of primary liver cancer and biliary cancer, which includes cancer of the gallbladder, limits application of mode-of-action data and may introduce misclassification bias. The recent Nordic cohort studies (Hansen 2004; Raaschou-Nielsen et al. 2003) disaggregate these cancers, and the addition of these two studies doubles the total number of epidemiologic studies providing information for primary liver cancer. The study by Raaschou-Nielsen et al. (2003), having greater statistical power because of its larger cohort size, suggested that both sites are possible targets of TCE toxicity, reporting a standardized incidence ratio (SIR) for primary liver cancer similar to that for gall-bladder and biliary tract cancer. Risks for the larger category of liver and biliary tract cancers are presented in both the Nordic studies and the two recent community studies (Lee et al. 2003; Morgan and Cassady 2002). These studies together suggest a modest association (risks between 1.1 and 2.8), with no clear pattern with duration of exposure. Furthermore, none of the studies have sufficient power to identify sex differences in susceptibility.

New information on lymphomas, including NHL and leukemia, and TCE exposure comes from cohort and community studies (Table 7). Both Nordic studies (Hansen et al. 2001; Raaschou-Nielsen et al. 2003) reported statistically significant associations with NHL, with increasing SIRs with increasing duration of employment. The risk of NHL mortality in Zhao et al. (2005) was more consistent than the NHL incidence with risks observed in Nordic cohorts. Except in the case of Raaschou-Nielsen et al. (2003), numbers of exposed NHL cases are small, limiting statistical power. The one available case–control study observed a strong but imprecise association between maternal exposure to TCE-contaminated drinking water during pregnancy and childhood leukemia (Costas et al. 2002). Aickin (2004) provides further evidence for an association between TCE in drinking water and childhood leukemia. Analyses using Bayesian statistical methods confirmed an elevated mortality in children from leukemia. Examining childhood leukemia incidence, Aickin (2004) reported that a rate ratio ≤ 1.0 was not credible, and risk > 2.0 could not be ruled out.

**Figure 1:**
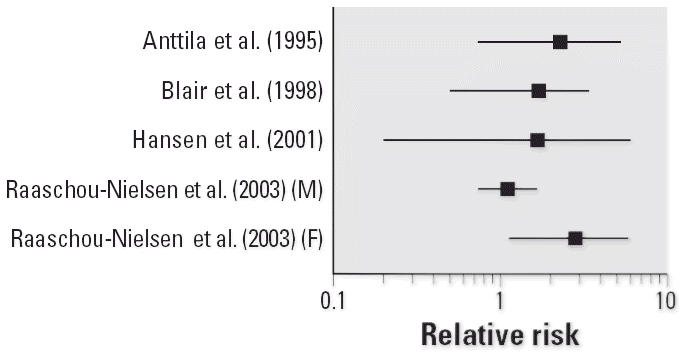
Relative risks (SIRs or SMRs) for primary liver cancer in occupational cohort studies of TCE-exposed workers. Abbreviations: F, female; M, male. No case–control studies of primary liver cancer and TCE exposure were identified from the published literature.

**Figure 2:**
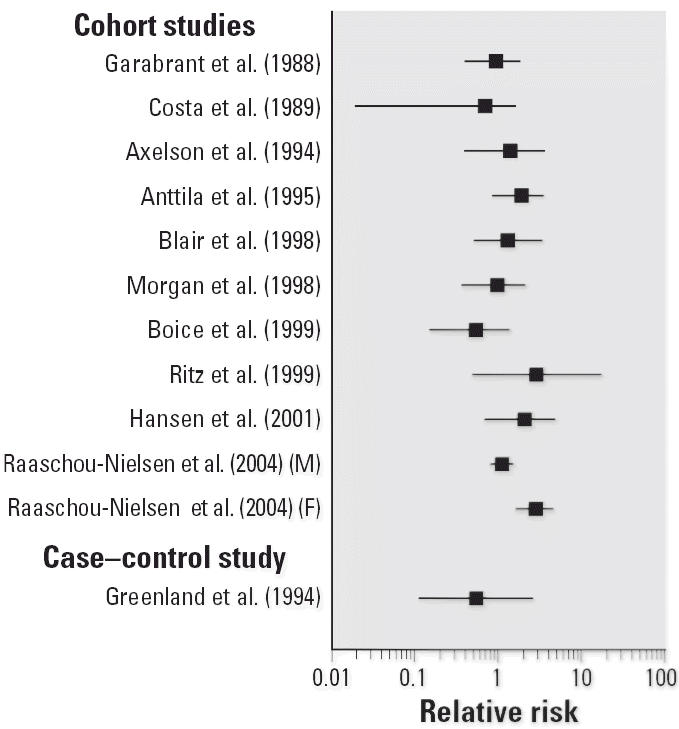
Relative risks for liver and biliary passage cancer in occupational cohort studies on TCE. SIRs or SMRs are presented for occupational cohort studies, and ORs for the case–control study.

**Figure 3:**
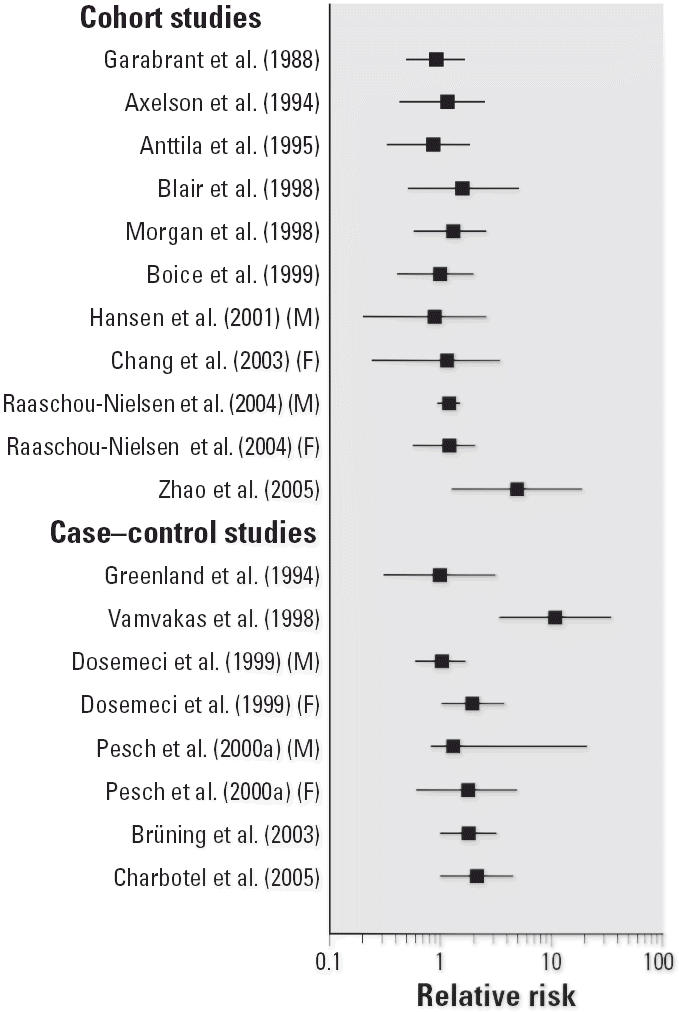
Relative risks for kidney cancer or RCC and TCE exposure. SIRs or SMRs are presented for occupational cohort studies, and ORs for case–control studies.

**Figure 4:**
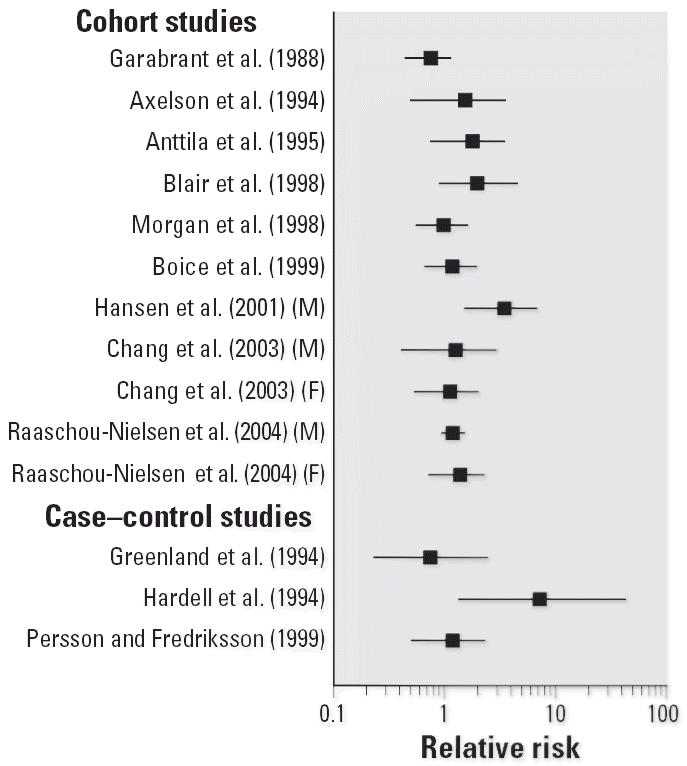
Relative risks for NHL and TCE exposure in cohort and case–control studies. SIRs or SMRs are presented for occupational cohort studies, and ORs for case–control studies.

To illustrate the potential impact of these new studies, Figures 1–4 show relative risks, SIRs, and standardized mortality ratios (SMRs) from cohort studies and ORs from case–control studies for four cancer sites discussed above (liver, liver and biliary passages, kidney, and NHL, respectively). These figures include studies published before 2000 [reviewed in, e.g., Wartenberg et al. (2000)] and those discussed above. The integration of this new information will contribute substantially to the hazard characterization of a TCE health evaluation and become an integral part of the U.S. EPA revised TCE risk assessment. However, this integration requires consideration of a number of key issues related to interpretation and synthesis, as discussed below.

## Issues Related to TCE Epidemiologic Evidence

### Studies of cancer incidence or cancer mortality

Both cancer incidence and cancer mortality rates are potentially useful in risk assessment for identifying hazards and assessing dose–response relationships. Incidence rates, generally considered to provide an accurate indication of risk of a disease in a population, are rarely available. In the absence of incidence data, epidemiologic studies have commonly relied on mortality data to assess exposure–disease associations. An understanding of the accuracy of death certificate information as a surrogate for incidence data is important for evaluating observations in the mortality studies. Known inaccuracies exist between cancer incidence and death certificate recordings for some cancer sites important to evaluating TCE exposure, for example, cancer of liver (primary) and liver and biliary passages (Percy et al. 1990). In their study of death certificate accuracy, Percy et al. (1990) showed that only 53% of 2,388 incident cases of primary liver cancer were actually attributed on the death certificate to this disease. Zhao et al. (2005) were able to examine both incidence and mortality among TCE-exposed workers and observed underreporting on death certificates for several site-specific cancers, including NHL, leukemia, and kidney and bladder cancers.

Death certificate inaccuracies would obscure exposure–disease associations toward the null by reducing statistical power and may explain apparent inconsistencies between epidemiologic studies using incidence data versus those based on death certifications. For example, apparent inconsistencies in some observations from cohort studies of American workers, which were primarily based on mortality, and cohort studies of Nordic workers, which were largely based on incidence, may reflect misclassification of death certificates compared with incidence data.

### Non-Hodgkin lymphoma

Lymphoma, including NHL, is a disease composed of numerous, etiologically distinct neoplasms (Fisher 2003; Herrinton 1998). Several issues may affect interpretation of NHL associations in the TCE epidemiologic studies and may be important to evaluating the consistency, or lack there of, across studies. First, epidemiologic studies evaluating NHL and TCE exposure have used a number of different *International Classification of Diseases* (ICD) revisions. All four Nordic studies (Anttila et al. 1995; Axelson et al. 1994; Hansen et al. 2001; Raaschou-Nielsen et al. 2003) classified NHL according to the seventh revision of the ICD [ICD-7; World Health Organization (WHO) 1957], and all reported consistent findings. Other revisions of the ICD were used in the more recent studies by Blair et al. (1998) [ICD Adapted (ICDA)-8, National Center for Health Statistics 1967], Boice et al. (1999) (ICD-9, WHO 1977), Garabrant et al. (1988) (ICD-9 in effect at date of death: ICD-7, ICDA-8, or ICD-9), Morgan et al. (1998, 2000) (ICD in effect at date of death: ICD-7, ICDA-8, or ICD-9), and Ritz (1999) (ICD-9). Few case–control studies on lymphoma are available. NHL cases in Hardell et al. (1994) were histologically verified and were classified using the Rappaport system. Persson et al. (1989) do not identify the system used to classify NHL cases in their study. Classification of lymphomas has changed with each revision.

Second, understanding of histopathologic and immunologic characteristics of lymphoma has grown since 1977, the publication date of ICD-9. Past classifications of lymphomas do not reflect the current biologic understanding of NHL and do not make distinctions between different cell types. From this perspective, lymphomas are defined broadly as B-cell and T-cell lymphomas, with further divisions into precursor neoplasms and mature neoplasms (Cogliatti and Schmid 2002). This implies that lymphomas classified in the past into distinct categories may share common biological properties and differentiation pathways. For example, a lymphoma of B-cell origin may be classified under older schemes as NHL, multiple myeloma, or leukemia. Emerging data on molecular markers of lymphoma suggest stage of cell differentiation at time of exposure as an important factor in NHL development (Staudt and Dave 2005).

### Exposure assessment issues in TCE epidemiologic studies

The methods by which exposure is assessed in epidemiologic studies of TCE are diverse, ranging from use of broad job or industry categories to analysis of biomonitoring data. Generally, greater weight is assigned to studies with more precise and specific exposure estimates. Careful evaluation of a study’s exposure assessment method is important in the evaluation of a body of epidemiologic data, particularly if divergent observations may be due to exposure misclassification bias reflecting incorrect assignment of study subjects to exposure groups. Many of the TCE studies lack actual exposure measurements for individual subjects, and surrogates such as available current or historical monitoring data are often used to reconstruct exposure parameters.

The three Nordic cohorts of Axelson et al. (1994), Anttila et al. (1995), and Hansen et al. (2001) identified study subjects using the TCE biological marker of urinary trichloroacetic acid (U-TCA), which provides some evidence of past TCE exposure, although usually not a full exposure history. These studies carry weight in the overall analysis because of their greater precision of exposure assessment compared with methods discussed below for other cohorts; however, a consideration of statistical power is also important because of fewer subjects compared with cohorts identified using other methods.

Other cohort and case–control studies have adopted a number of approaches for exposure assessment. TCE exposure has been assigned to subjects using surrogate information based on patterns of TCE use by job title obtained from historical job descriptions, from historical industrial hygiene surveys, or from personal interviews to develop job exposure matrices (JEMs). For several cohorts, industrial hygiene measurements either were absent before the 1970s (Boice et al. 1999; Marano et al. 2000; Morgan et al. 1998, 2000) or were quite limited (Blair et al. 1998; Stewart et al. 1991). Furthermore, some cohort (Ritz 1999) and case–control (Greenland et al. 1994) studies classified study subjects as TCE exposed using information obtained from personal interviews or generic JEMs or job-task exposure matrices (JTEMs) in the absence of historical monitoring. Two issues associated with the use of generic JEMs are sensitivity (i.e., the ability to identify study subjects as exposed) and specificity (i.e., the ability to identify study subjects as not exposed).

Still other cohort studies (Chang et al. 2003, 2005; Costa et al. 1989; Garabrant et al. 1988) have defined exposure using occupation and industry. TCE is identified as one of a number of potential exposures, but no information is provided on individual subjects with TCE exposure. The main shortcoming of this type of study is that the lack of an association with a particular job or industry may mask the effect of exposure to a specific chemical to which only some individuals in the job are exposed (Teschke et al. 2002). For this reason, a consideration of potential exposure misclassification bias is important in weighting these studies in an overall weight of evidence.

In addition, multiple solvents and chemical agents are common in the TCE studies, adding to the complexity of exposure assessment and inferences about causality. Some studies of TCE also identify exposures to other chlorinated solvents such as perchloroethylene and 1,1,1-trichloroethane (Blair et al. 1998; Boice et al. 1999; Marano et al. 2000; Morgan et al. 1998, 2000; Stewart et al. 1991; Zhao et al. 2005). The potential for exposure to multiple chlorinated solvents is an important consideration in the TCE epidemiologic studies for two reasons. First, these chemicals can share similar metabolic profiles or modes of action as TCE (U.S. EPA 2001), and second, some epidemiologic studies have also reported independent associations between exposure to these other solvents and cancer (Blair et al. 1998; Zhao et al. 2005). Physiologically based pharmacokinetic models such as those discussed by Chiu et al. (2006b) may be useful for better understanding cumulative exposure in these epidemiologic studies.

## Approaches for Causal Inference

The practice of causal inference in environmental epidemiology relies on three approaches: narrative reviews, criteria-based inference methods, and, increasingly, meta-analysis (Weed 2002). All three have been employed in various analyses of the epidemiologic literature on cancer and TCE exposure. Narrative reviews of a body of epidemiologic evidence generally do not fully consider potential biases and confounding factors. By contrast, criteria-based approaches for assessing causality evaluate evidence according to a set of criteria or standards applied to the evidence (Weed 2002). For instance, the aspects proposed by Sir Bradford Hill (1965) are widely cited for framing the factors to consider in determining whether statistical associations are likely to be causal. Similar criteria are also presented in the U.S. EPA *Guidelines for Carcinogen Risk Assessment* (U.S. EPA 2005).

Criteria-based approaches have increasingly been supplemented with formal statistical methods such as meta-analysis for reviewing and summing a body of evidence (Weed 2002). Common meta-analytic methods can include fitting of fixed-effects or random-effects models, linear regression analysis to assess dose–response, or pooled analyses. Pooled analysis of the Nordic studies may be more feasible because of their similar design and similar follow-up period for documenting cancer incidence than for other TCE cohorts. As discussed in the overview article of this mini-monograph by Chiu et al. (2006a), the NAS has been asked to provide advice on appropriate meta-analysis methods, including the classification and weighting of individual studies.

## Discussion and Summary

The U.S. EPA draft TCE assessment (U.S. EPA 2001) noted that epidemiologic studies, when considered as a whole, have associated TCE exposure with excess risk of kidney, liver, lymphohematopoietic, cervical, and prostate cancer. Recently published studies appear to provide further support for several of those conclusions, suggesting, as do previous studies, modestly elevated site-specific risk (typically between 1.5 and 2.0), given exposure conditions in the epidemiologic studies.

The recent epidemiologic studies strengthen the evidence that the kidney is a target of TCE toxicity. It should be noted that kidney toxicity besides cancer has been found by Radican et al. (2006), who reported a statistically significant association with end-stage renal disease mortality and exposure to solvents, including TCE. Understanding the mechanism by which TCE may act in kidney toxicity, including cancer, can inform cause–effect evaluations. The glutathione *S*-transferase (GST) metabolic pathway has been hypothesized as important to mode-of-action considerations (Caldwell and Keshava 2006), and GST polymorphisms are reported to influence RCC risk associated with TCE exposure (Brüning et al. 1997). Brauch et al. (2004) examined somatic mutation to the von Hippel-Lindau tumor suppressor gene in renal cell tumors of non-TCE-exposed cases, comparing the prevalence of mutation to that found in renal tumors of TCE-exposed subjects reported in an earlier publication (Brauch et al. 1999). A higher prevalence of somatic mutations was found in renal cell tumors of TCE-exposed cases than in tumors of non-TCE-exposed cases. Moreover, the C > T transition at nucleotide 454, detected in some RCCs from TCE-exposed subjects, was not found among the non-TCE-exposed RCC cases.

The recent studies also support the liver and immune system as being targets for TCE toxicity, with most of these studies showing elevated (and in some cases statistically significant) cancer risks from TCE exposure. However, although the number of studies assessing primary liver cancer separately from biliary tract cancers has doubled, the total number is still only 4, compared to 11 examining the combined category. With lymphomas, there are also a number of classification issues, including the use of different ICD revisions, and the fact that these groupings may lump together etiologically distinct neoplasms. Moreover, studies evaluating these end points include both incidence and mortality studies, which may have different sensitivity and biases. Thus, the reduced specificity in most studies, in combination with the relatively small number of total cases due to low background incidence, complicates interpretation of these findings.

Of particular importance for assessment of epidemiologic evidence on TCE exposure is characterizing the totality of the evidence in light of factors that may contribute to false positive findings or to false negative observations. The evidence presented on issues regarding data sources, exposure assessment, and disease classification can influence the statistical power of the epidemiologic study to detect whether there is an underlying risk. The challenge is to consider these issues, along with well-articulated approaches when evaluating the body of evidence, including the application of meta-analysis methods and rationale for grouping individual studies, in identifying hazards and drawing causal conclusions.

## Figures and Tables

**Table 1:** Occupational cohort studies of cancer and TCE exposure.

Reference	Description	Size of study and comparison group	Exposure assessment
Aircraft and aerospace workers			
Zhao et al. 2005	Aerospace workers with at least 2 years of employment at Boeing/Rockwell/Rocketdyne (Santa Susana Field Laboratory, Ventura, CA) between 1950 and 1993. Cancer mortality as of 31 December 2001.	6,044 (2,689 with high cumulative exposure to TCE). Mortality rates of subjects in lowest TCE exposure category.	Industrial hygienist assessment from walk-through visits, interviews, and review of historical facility reports. Each job title ranked for presumptive TCE exposure as high (3), medium (2), low (1), or no (0) exposure. Cumulative TCE assigned to individual subjects using JEM. Exposure–response patterns assessed using cumulative exposure.
	Aerospace workers with at least 2 years of employment at Boeing/Rockwell/Rocketdyne (Santa Susana Field Laboratory) between 1950 and 1993 who were alive as of 1988. Cancer incidence was ascertained between 1988 and 2000.	5,049 (2,227 with high cumulative exposure to TCE). Incidence rates of subjects in lowest TCE exposure category.	
Cohorts identified from U-TCA			
Hansen et al. 2001	Workers biologically monitored for occupational exposure to TCE between 1947 and 1989 using U-TCA and air TCE measurements between 1947 and 1989 and alive as of 1 April 1968. Follow-up for cancer incidence from 1 April 1968 or date of first employment through 31 December 1996.	803 (16,703 P-Y). Cancer incidence rates of the Danish population.	Of the 803 subjects, 712 had U-TCA, 89 had air TCE measurement records and 2 had records of both types. Median TCE concentration was 19 mg/m^3^. Mean and median concentrations of U-TCA were 250 μmol/L and 92 μmol/L, respectively. There were on average 2.2 U-TCA measurements per individual.
Other cohorts
Chang et al. 2005	Workers employed between 1978 and 31 December 1998 at an electronics factory in Taiwan. Follow-up began on 1 January 1979 or date of entry to the cohort through 31 December 1997. Cancer incidence ascertained as of 31 December 1997.	86,868 (1,380,355, P-Y). Incidence rates of Taiwanese population.	National Labor Department inspection reports and the company’s import/export statistics indicated use of many chlorinated solvents, including TCE, in the manufacturing process. No information on TCE use, potential TCE exposure concentrations, or the percentage of study subjects whose job titles indicated potential TCE exposure.
Chang et al. 2003	Workers employed between 1978 and 31 December 1997 at an electronics factory in Taiwan. Follow-up began on 1 January 1985 or date or entry to the cohort through 31 December 1997. Vital status ascertained from 1 January 1985 through 31 December 1997.	86,868 (1,380,355 P-Y) .Mortality rates of Taiwanese population.	
Raaschou-Nielsen et al. 2003	Blue-collar workers employed between 1964 and 1997 for at least 3 months and alive as of 1 January 1968 at 347 Danish TCE-using companies. Follow-up for cancer incidence from 1 April 1968 or date of first employment through 31 December 1997.	40,049 (14,360 with presumably higher level exposure to TCE) (339,486 P-Y). Cancer incidence rates of the Danish population.	Employers had documented TCE use. Blue-collar versus white-collar workers and companies with ≤ 200 workers were variables identified as increasing the likelihood for TCE exposure. Subjects were identified from the following industries: iron and metal, electronics, painting, printing, chemical, and dry cleaning.

Abbreviations: JEM, job exposure matrix; P-Y; person-years; U-TCA, urinary trichloroacetic acid.

**Table 2:** Case–control epidemiologic studies examining cancer and TCE exposure.

Reference	Population	Cases (no.)	Controls (no.)	Response rate (%)	Exposure assessment	Statistical analysis
Brain (neuroblastoma)						
DeRoos et al. 2001	Cases in children ≤19 years of age selected from Children’s Cancer Group and Pediatric	504	504	Cases, 73 Controls, 74	Telephone interview with parent using questionnaire to assess parental occupation and self-reported exposure history and judgment-based attribution of exposure to TCE and other solvents.	Logistic regression with covariate for child’s age and material race, age, and education.
Olshan et al. 1999	Oncology Group with diagnosis in 1992–1994; population controls (random digit dialing) matched to control for birth date.					
Rectal						
Dumas et al. 2000	Male cases, 35–70 years of age, diagnosed in 1979–1985 and histologically confirmed; controls with cancers at other sites chosen from same cancer registry as cases (group 1) or population controls (group 2).	257	1,295 (group 1) 533 (group 2)	Cases, 85 Controls, 100 (group 1) Controls, 72 (group 2)	In-person or telephone interview to assess self-reported occupational history: TCE exposure assigned to subject using work history obtained by interview and JEM.	Logistic regression analyses adjusted for age, education, cigarette smoking, beer consumption, body mass index,and respondent status.
Renal cell						
Brüning et al. 2003	Histologically confirmed cases from German hospitals (Arnsberg) in 1992–2000; controls frequency-matched (one case, three controls) by sex and age to cases, from hospitals with urology department (and local geriatric department for older controls) serving Arnsberg.	134	401	Cases, 83 Controls, no information	In-person interview with case or next-of-kin; questionnaire assessing occupational history using job title and JEM of Pannett et al. (1985).	Logistic regression with covariates for age, sex, and smoking.
Charbotel et al. 2005, 2006 Fevotte et al. 2006	Histologically confirmed cases from three hospitals and urologists in the High Savoy area and surrounding region in France and from Geneva, Switzerland, in 1993–2003; controls selected from urologists’ files matched 1:4 to case for birth year and sex.	86	316	Cases, 74 Controls, 78	Blinded telephone interview with case or next-of-kin; questionnaire assessing occupational history using JTEM or self-reported exposure to assign TCE and other exposures.	Matched pairs conditional logistic regression with covariates for body mass index and tobacco smoking.
Pesch et al. 2000a	Histologically confirmed cases from German hospitals (five regions) in 1991–1995; controls randomly selected from residency registries matched for region, sex, and age.	935	4,298	Cases, 88 Controls, 71	In-person interview with case or next-of-kin; questionnaire assessing occupational history using job title or self-reported exposure to assign TCE and other exposures.	Logistic regression with covariates for age, family income, ethnicity, smoking, and respondent status.
Urothelial						
Pesch et al. 2000b	Histologically confirmed cases from German hospitals (five regions) in 1991–1995; controls randomly selected from residency registries matched for region, sex, and age.	1,035	4,298	Cases, 84 Controls, 71	In-person interview with case or next-of-kin; questionnaire assessing occupational history using job title or self-reported exposure to assign TCE and other exposures.	Logistic regression with covariates for age, family income, ethnicity, smoking, and respondent status.

JTEM, job-task exposure matrix.

**Table 3:** Community studies on cancer and TCE exposure.

Reference	Description	Statistical methods	Exposure assessment
Ahrens et al. 2001 Waller and Turnbull 1993 Waller et al. 1992 Turnbull et al. 1990	Incident leukemia cases from 1978–1982 from eight counties in upstate New York.	Illustration of three statistical methodologies to assess clustering of leukemia cases and 12 hazardous waste sites.	Residence in census tract or census block group with a previously identified inactive hazardous waste site.
Aickin 2004 Aickin et al. 1992 Flood et al. 1990, 1997 Flood and Chapin 1988	Deaths due to cancer, including leukemia, congenital anomalies, injuries, and cardiovascular diseases in 1966–1986 and childhood leukemia incident cases (1965–1986) among residents of Maricopa County, Arizona.	Standardized rate ratios for mortality from Poisson regression modeling. Childhood leukemia incidence data evaluated using Bayes methods and Poisson regression modeling.	Resident of Maricopa County, AZ, at the time of diagnosis or death as surrogate for exposure.
Costas et al. 2002, Massachusetts Department of Public Health 1997	Childhood leukemia (≤19 years age) diagnosed in 1969–1989 in residents of Woburn, MA; controls randomly selected from Woburn public school records, matched for age.	Logistic regression with composite covariate, a weighted variable of individual covariates.	Questionnaire administered to parents separately assessing demographic and lifestyle characteristics, medical history information, environmental and occupational exposure, and use of public drinking water in the home. Hydraulic mixing model used to infer drinking water containing TCE and other solvents delivered to residence.
Lee et al. 2003	Cancer deaths in 1966–1997 in two villages in Taiwan; controls were cardiovascular and cerebrovascular disease deaths from same underlying area as cases.	Mortality OR using Mantel-Haenszel method and stratified by gender and age and logistic regression with covariates for age and period.	Location of residence as recorded on death certificate. Monitoring in 1999–2000 of TCE in groundwater or well water was used to infer exposure to TCE to village residents.
Morgan and Cassady 2002	Cancer cases diagnosed between 1 April 1988 and 31 December 1998 among residents of 13 census tracts in Redlands area, San Bernardino County, CA.	Standardized incidence rates for all cancer sites and 16 site-specific cancers; expected numbers of cancers using incidence rates of site-specific cancer of a four-county region in 1988–1992.	TCE and perchlorate detected in some county wells; no information on distribution of contaminated water to residents. TCE concentrations in water after 1991 were below maximum contaminant level of 5 ppb.

OR, odds ratio.

**Table 4:** Select epidemiologic studies: site-specific cancer and exposure to TCE.

Reference	Study population	Exposed cases (no.)	Estimated relative risk (95% CI)
Total cancer			
Cohort studies			
Hansen et al. 2001	Male	109	1.0 (0.9–1.3)
	Female	19	1.0 (0.6–1.6)
Chang et al. 2003	Male	66	0.7 (0.5–0.8)
	Female	250	1.0 (0.9–1.1)
Raaschou-Nielsen et al. 2003	Male	2,434	1.1 (1.0–1.1)
	Female	624	1.2 (1.1–1.3)
Community studies			
Lee et al. 2003	Upstream village	266^a^	1.0
	Downstream village		2.1 (1.3–3.3)^b^
Morgan and Cassady 2002	13 census tracts in San Bernardino County, CA	3,098	1.0 (0.9–1.0)
Bladder			
Cohort studies			
Hansen et al. 2001	Male	10	1.1 (0.5–2.0)
	Female	0	
Chang et al. 2003	Male	1	1.0 (0.01–5.4)
	Female	1	1.0 (0.01–5.4)
Raaschou-Nielsen et al. 2003	Male	203	1.0 (0.9–1.2)
	Female	17	1.6 (0.9–2.6)
Zhao et al. 2005^c^	Low TCE score	7	1.0
	Medium TCE score	7	1.5 (0.8–2.9)
	High TCE score	3	2.0 (0.9–4.2)
Case–control studies			
Pesch et al. 2000b	JTEM, male		
	Medium TCE exposure	47	0.8 (0.6–1.2)
	High TCE exposure	74	1.3 (0.9–1.7)
	Substantial TCE exposure	36	1.8 (1.2–2.7)
Community studies			
Morgan and Cassady 2002	13 census tracts in San Bernardino County, CA	82	1.0 (0.8–1.2)
Breast			
Cohort studies			
Hansen et al. 2001	Female	4	0.9 (0.2–2.3)
Chang et al. 2005	Female	215	1.2 (1.0–1.4)
Raaschou-Nielsen et al. 2003	Male	2	0.5 (0.1–1.9)
	Female	145	1.1 (0.9–1.2)
Community studies			
Morgan and Cassady 2002	Females in 13 census tracts in San Bernardino County, CA	536	1.1 (1.0–1.2)
Esophagus			
Cohort studies			
Hansen et al. 2001	Male	6	4.2 (1.5–9.2)
	Female	0	
Chang et al. 2005	Male	0	
	Female	0	
Raaschou-Nielsen et al. 2003	Male	23^d^	1.8 (1.2–2.7)
	Female	0	
Zhao et al. 2005^c,e^	Low TCE score	7	1.0
	Medium TCE score	7	1.7 (0.6–4.4)
	High TCE score	3	1.3 (0.2–4.0)

CI, confidence interval.

a Total cancer deaths in the two villages.

b 99% CI.

c Zhao et al. (2005) present both cancer incidence and cancer mortality. Relative risks in this table are for cancer incidence.

d Adenocarcinoma of the esophagus.

e Esophageal and stomach cancer incidence.

**Table 5:** Select epidemiologic studies: kidney or renal cell cancer and exposure to TCE.

Reference	Study population	Exposed cases (no.)	Estimated relative risk (95% CI)
Cohort studies			
Hansen et al. 2001	Male	3	0.9 (0.2–2.6)
	Female	1	2.4 (0.03–14)
Chang et al. 2005	Male	0	
	Female	3	1.2 (0.2–3.4)
Raaschou-Nielsen et al. 2003	Male	93	1.2 (1.0–1.5)
	Female	10	1.2 (0.6–2.1)
	Duration of employment, male		
	≤ 1 year	14	0.8 (0.5–1.4)
	1–4.9 years	25	1.2 (0.8–1.7)
	≥ 5 years	29	1.6 (1.1–2.3)
	Duration of employment, female		
	≤ 1 year	2	1.1 (0.1–3.8)
	1–4.9 years	3	1.2 (0.2–3.5)
	≥ 5 years	3	1.5 (0.3–4.3)
Zhao et al. 2005^a^	Low TCE score	6	1.0
	Medium TCE score	6	1.9 (0.6–6.2)
	High TCE score	4	4.9 (1.2–20)
Case-control			
Pesch et al. 2000a	JTEM, male		
	Medium exposure	68	1.3 (1.0–1.8)
	High exposure	59	1.1 (0.8–1.5)
	Substantial exposure	22	1.3 (0.8–2.1)
	JTEM, female		
	Medium exposure	11	1.3 (0.7–2.3)
	High exposure	7	0.8 (0.4–1.9)
	Substantial exposure	5	1.8 (0.6–5.0)
Brüning et al. 2003	Employment in industry with TCE exposure	117	1.8 (1.2–2.7)
	Self-assessed, TCE	25	2.5 (1.4–4.5)
	Duration of exposure		
	No exposure	109	1.0
	≤ 10 years	14	3.8 (1.5–9.3)
	10– ≤ 20 years	13	1.8 (0.7–4.8)
	20+ years	6	2.7 (0.8–8.7)
Charbotel et al. 2005, 2006	Cumulative TCE dose		
	Nonexposed	49	1.0
	Low	12	1.6 (0.8–3.5)
	Medium	9	1.2 (0.5–2.8)
	High	16	2.2 (1.0–4.6)
	Cumulative TCE dose + peaks		
	Nonexposed	49	1.0
	High + peaks	8	2.7 (1.1–7.1)
Community studies			
Morgan and Cassady 2002	13 census tracts in San Bernardino County, CA	54	0.8 (0.6–1.1)

a Zhao et al. (2005) present both cancer incidence and cancer mortality. Relative risks in this table are for cancer incidence.

**Table 6:** Select epidemiologic studies: liver cancer and exposure to TCE.

Reference	Study population	Exposed cases (no.)	Estimated relative risk (95% CI)
Liver, primary			
Cohort studies^a^			
Hansen et al. 2001			
Hansen 2004	Male, female	2	1.7 (0.2–6.0)
Chang et al. 2003	Male	0	
	Female	0	
Raaschou-Nielsen et al. 2003	Male	27	1.1 (0.7–1.6)
	Female	7	2.8 (1.1–5.8)
	Duration of employment, male		
	≤ 1 year	9	1.3 (0.6–2.5)
	1–4.9 years	9	1.0 (0.5–1.9)
	≥ 5 years	9	1.1 (0.5–2.1)
	Duration of employment, female		
	≤ 1 year	2	2.8 (0.3–10)
	1–4.9 years	4	4.1 (1.1–11)
	≥ 5 years	1	1.3 (0.0–7.1)
Liver and bile ducts			
Cohort studies^a^			
Hansen et al. 2001			
Hansen 2004	Male and female	5	2.1 (0.7–4.9)
Chang et al. 2003	Not reported		
Raaschou-Nielsen et al. 2003	Males	41	1.1 (0.8–1.5)
	Females	16	2.8 (1.6–4.5)
	Duration of employment, male		
	≤ 1 year	13	1.2 (0.6–2.1)
	1–4.9 years	13	0.9 (0.5–1.6)
	≥ 5 years	15	1.1 (0.6–1.7)
	Duration of employment, female		
	≤ 1 year	4	2.5 (0.7–6.4)
	1–4.9 years	10	4.5 (2.1–8.3)
	≥ 5 years	2	1.1 (0.1–3.8)
Community studies			
Lee et al. 2003	Upstream village	53^b^	1.0
	Downstream village		2.6 (1.2–5.5)
Morgan and Cassady 2002	13 census tracts in San Bernardino County, CA	28	1.3 (0.9–1.9)

a Zhao et al. (2005) did not present relative risks for liver or liver and bile duct cancer in their article.

b Total liver cancer deaths in the two villages.

**Table 7:** Select epidemiologic studies: lymphoma and exposure to TCE.

Reference	Study population	Exposed cases (no.)	Estimated relative risk (95% CI)
NHL			
Cohort studies			
Hansen et al. 2001	Male	8	3.5 (1.5–6.9)
	Female	0	
	Duration of employment, male		
	Unknown	2	3.7 (0.4–13)
	≤ 6.25 years	2	2.5 (0.3–9.2)
	≥ 6.25 years	4	4.2 (1.1–11)
Chang et al. 2005	Male	5	1.3 (0.4–3.0)
	Female	10	1.1 (0.6–2.1)
Raaschou-Nielsen et al. 2003	Male	83	1.2 (1.0–1.5)
	Female	13	1.4 (0.7–2.3)
	Duration of employment, male		
	≤ 1 year	23	1.1 (0.7–1.6)
	1–4.9 years	33	1.3 (0.9–1.8)
	≥ 5 years	27	1.4 (0.9–2.0)
	Duration of employment, female		
	≤ 1 year	2	0.7 (0.1–2.4)
	1–4.9 years	6	1.6 (0.6–3.5)
	≥ 5 years	5	1.8 (0.6–4.3)
Zhao et al. 2005^a^	Low TCE score	28	1.0
	Medium TCE score	16	0.9 (0.5–1.7)
	High TCE score	1	0.2 (0.03–1.5)
Community studies			
Morgan and Cassady 2002	13 census tracts in San Bernardino County, CA	111	1.1 (0.9–1.3)
Leukemia			
Cohort studies			
Hansen et al. 2001	Male	5	1.9 (0.6–4.4)
	Female	1	3.1 (0.04–18)
Chang et al. 2005	Male	2	0.4 (0.05–1.6)
	Female	8	0.5 (0.2–1.1)
Raaschou-Nielsen et al. 2003	Male	69	1.1 (0.8–1.4)
	Female	13	1.7 (0.9–2.9)
Community studies			
Costas et al. 2002	Exposed to water from TCE-contaminated wells G and H 2 years before pregnancy to leukemia diagnosis		
	Never	3	1.0
	Least	9	5.0 (0.7–34)
	Most	7	3.6 (0.5–25)
	Exposed to water from TCE-contaminated wells G and H during pregnancy		
	Never	9	1.0
	Least	3	3.5 (0.2–58)
	Most	7	14 (0.9–224)^b^
Morgan and Cassady 2002	13 census tracts in San Bernardino County, CA	77	1.0 (0.8–1.3)

a Zhao et al. (2005) present both cancer incidence and cancer mortality. Relative risks in this table are for NHL and leukemia incidence combined.

b Test for trend is statistically significant, *p* ≤ 0.05.
